# Localization and Identification of Brain Microstructural Abnormalities in Paediatric Concussion

**DOI:** 10.3389/fnhum.2021.657374

**Published:** 2021-05-31

**Authors:** David Stillo, Ethan Danielli, Rachelle A. Ho, Carol DeMatteo, Geoffrey B. Hall, Nicholas A. Bock, John F. Connolly, Michael D. Noseworthy

**Affiliations:** ^1^Imaging Research Centre, St. Joseph’s Healthcare, Hamilton, ON, Canada; ^2^McMaster School of Biomedical Engineering, McMaster University, Hamilton, ON, Canada; ^3^School of Rehabilitation Sciences, McMaster University, Hamilton, ON, Canada; ^4^Department of Psychology, Neuroscience & Behaviour, McMaster University, Hamilton, ON, Canada; ^5^ARiEAL Research Centre, McMaster University, Hamilton, ON, Canada; ^6^Department of Linguistics, McMaster University, Hamilton, ON, Canada; ^7^Department of Electrical and Computer Engineering, McMaster University, Hamilton, ON, Canada; ^8^Department of Radiology, McMaster University, Hamilton, ON, Canada

**Keywords:** concussion, diffusion tensor imaging (DTI), paediatric, MRI, personalized

## Abstract

In the United States, approximately 2.53 million people sustain a concussion each year. Relative to adults, youth show greater cognitive deficits following concussion and a longer recovery. An accurate and reliable imaging method is needed to determine injury severity and symptom resolution. The primary objective of this study was to characterize concussions with diffusion tensor imaging (DTI). This was performed through a normative *Z*-scoring analysis of DTI metrics, fractional anisotropy (FA), axial diffusivity (AD), and radial diffusivity (RD), to quantify patient-specific injuries and identify commonly damaged brain regions in paediatric concussion patients relative to healthy controls. It was hypothesized that personalizing the detection analysis through normative *Z*-scoring would provide an understanding of trauma-induced microstructural damage. Concussion patients were volunteers recruited from the Emergency Department of the McMaster Children’s Hospital with a recent concussion (*n* = 26), 9 males and 17 females, mean age 14.22 ± 2.64, while healthy paediatric brain DTI datasets (25 males and 24 females, mean age 13.52 ± 1.03) were obtained from an MRI data repository. Significant abnormalities were commonly found in the longitudinal fasciculus, fronto-occipital fasciculus, and corticospinal tract, while unique abnormalities were localized in a number of other areas reflecting the individuality of each child’s injury. Total injury burden, determined by the number of regions containing outliers per DTI metric per patient, was used as the metric to quantify the overall injury severity of each patient. The primary outcome of this analysis found that younger patients experienced a significantly greater injury burden when measured using fractional anisotropy (*p* < 0.001). These results show that DTI was able to detect microstructural changes caused by concussion, on a per-person basis, and has the potential to be a useful tool for improving diagnostic accuracy and prognosis of a concussion.

## Introduction

Concussions are mild traumatic brain injuries (mTBI) that account for 70–90% of all TBI (Cassidy et al., 2004). In 2014 the Center for Disease Control and Prevention (CDC) recorded approximately 2.53 million TBI-related Emergency Department visits, with 812,000 resulting from children (The Centers for Disease Control and Prevention, 2020). In most cases symptoms resolve in a matter of days, however, youth show greater cognitive deficits following concussion and a longer recovery relative to adults (Zuckerman et al., 2012). Cognitive impairment resulting from a concussion can include memory, information processing speed, attention, and executive function (Moore et al., 2016). The safe and timely return to school and recreation is important for the physical and psychosocial development of children and adolescents affected by concussion.

Currently, subtle changes in brain structure caused by diffuse axonal injury (DAI), indicative of a concussion, are not detected using clinical magnetic resonance imaging (MRI) and computed tomography (CT) scans (Chamard and Lichtenstein, 2018). Therefore, it is paramount that reliable methods be developed to determine a safe return to sport or activity for each individual. Advanced imaging methods are required to improve diagnosis and prognosis in concussion cases. MRI techniques such as diffusion tensor imaging (DTI) are sensitive to subtle changes in white matter (WM) fiber tracts revealing microstructural DAI (Asken et al., 2018). In WM water diffusion is anisotropic, meaning the diffusivity is not equal in all directions. Utilizing tensors, the shape of the diffusion ellipsoid can be modeled by a parameter called fractional anisotropy (FA), which scales between zero (isotropic diffusion) and one (anisotropy). Higher FA is interpreted to be related to healthy, intact myelin. The tensor calculation provides further metrics of water mobility known as axial diffusivity (AD) and radial diffusivity (RD; Asken et al., 2018). DTI has been useful in understanding damaged myelin for a number of neurological disorders such as brain tumors, neurodegenerative dementia, and multiple sclerosis (Filippi and Agosta, 2016), but is not typically used clinically for concussion assessment. However, because a concussion causes axonal shearing damage (Asken et al., 2018), DTI could be a useful diagnostic tool.

Concussions are a unique injury where the individualization of grading and diagnosis is critically important. Comparison of an individual against a standardized reference sample of healthy age/sex-matched brains, using a *Z*-scoring approach, can be used to localize abnormalities in any single brain. A *Z*-score identifies outliers, relative to a normative dataset, and hence is hypothesized to indicate a region of damage while accounting for normal anatomical variability. Although DTI studies have been successful in adults, most only focus on FA as a metric rather than considering RD and AD (Asken et al., 2018). Furthermore, none have reported on statistical normality, necessary for valid *Z*-scoring, or the severity of clinical presentation. Thus, the goals of this study were to: (i) develop a *Z*-scoring approach for assessment of paediatric concussion, to validate the assumption of normality in *Z*-scoring; (ii) to determine whether *Z*-scoring of other DTI metrics (AD and RD) provide diagnostic utility in the personalized assessment of concussion; and (iii) to assess whether *Z*-score correlates with concussion severity.

## Materials and Methods

### Participants

Twenty-six subjects [nine males and 17 females, age = 14.22 ± 2.64 years; average ± standard deviation (S.D.)] recently diagnosed with a concussion were recruited from the emergency department of the McMaster Children’s Hospital, 13 of which sustained a sport-related concussion (Table 1). The study was approved by the Hamilton Integrated Research Ethics Board (HiREB), with informed consent obtained from a parent or guardian, and assent obtained from the youth subject. At the time of scanning, participants were graded on symptom severity using the Post-Concussion Symptom Scale (PCSS; Lovell et al., 2006). Data from 49 healthy, age-matched controls [25 males and 24 females, age = 13.52 ± 1.03 years; average ± standard deviation (S.D.)] was obtained from the *Paediatric MRI Data Repository* created by the *NIH MRI Study of Normal Brain Development* (Rivkin et al., 2016).

**Table 1:** Demographics per participant with regard to their current and previous concussion(s).

Participant	Age at injury	Number of previous concussions	Loss of consciousness	Time from injury to MRI scan (days)	PCSS
1	9.63	0	Yes	142	71
2	13.65	1	Unknown	11	48
3	11.79	0	Yes	10	56
4	10.07	0	No	17	62
5	15.03	0	No	19	76
6	10.8	0	No	14	36
7	15.42	1	No	15	77
8	16.99	1	No	185	64
9	14.2	0	No	15	82
10	16.86	0	Yes	27	19
11	15.92	0	No	196	47
12	14.8	0	No	33	52
13	10.38	1	No	146	2
14	16.3	0	Unknown	20	22
15	11.61	2	No	31	39
16	17.4	4	Unknown	79	62
17	16.4	2	No	21	36
18	16.65	2	No	98	42
19	12.3	0	No	37	56
20	16.67	0	Yes	27	6
21	15.21	1	Unknown	27	90
22	10.44	1	Yes	28	20
23	10.34	0	Unknown	71	26
24	15.31	1	No	57	47
25	14.43	1	No	38	84
26	16.9	3	No	195	52

### Data Acquisition and Pre-processing

Imaging was done using a 3 Tesla GE Discovery MR750 MRI system and 32-channel head and neck coil (General Electric Healthcare, Milwaukee, WI). Following a 3-plane localizer, a 3D inversion recovery (IR) prepped fast spoiled gradient recalled echo (fSPGR) T1-weighted scan was performed (TE/TR/TI = 4.3/11.4/450 ms, flip angle = 12°, 512 × 256 matrix, 140 slices, 24 cm field of view (FOV), reconstructed to 1 mm^3^ isotropic voxels).

The DTI spin-echo, echo-planar imaging (SE-EPI) protocol was split into three separate scans of 19-, 20-, and 21-directions (each with four *b* = 0 s/mm^2^ images) to permit motion and eddy current correction on smaller blocks of data during the analysis pipeline. The three scans were then concatenated to form a single merged 60-direction DTI dataset containing 12 *b* = 0 s/mm^2^ and 60 *b* = 1,000 s/mm^2^ volumes for a total of 72 images per slice (Jakab et al., 2017). For each block of diffusion images (TE/TR = 87/10,000 ms, 3 mm isotropic voxels) MRI pre-scan values were kept constant. The DTI scans for the downloaded control dataset were performed on the same MRI systems with identical resolution and similar diffusion parameters. A B_0_ field map was also acquired with identical acquisition geometry to the DTI scan.

After image concatenation, processing was performed using FSL (Smith et al., 2004; Jenkinson et al., 2012). Initial steps included eddy current correction, motion correction, and B_0_ field distortion correction through affine registration to a reference volume using the FSL tool ‘***eddy***’ (Andersson and Sotiropoulos, 2016). Afterward, the FSL Brain Extraction Tool (*BET*) was applied to remove the skull and optic nerves to produce a binary brain mask (Smith, 2002). The diffusion tensors were then reconstructed using the default standard linear regression algorithm of ***dtifit*** in FSL (Behrens et al., 2003, 2007). The final output images from ***dtifit*** included FA, eigenvalue (λ_1_, λ_2_, λ_3_), and eigenvector (ε_1_, ε_2_, ε_3_) images, where AD = λ_1_ and RD was computed from (λ_2_+λ_3_)/2. All control brains were processed in a similar fashion.

All brains, controls and subjects, were registered to the MNI152 1 mm T1-weighted average structural template using the FSL utility ***flirt*** (Jenkinson and Smith, 2001; Jenkinson et al., 2002; Greve and Fischl, 2009). More specifically a 12 degree of freedom (df) trilinear affine transformation was applied to register the FA maps to the standard atlas (with all other DTI metrics following). Within FSLView, two brain atlases were used to obtain 24 unique regions of interest (ROI) from the Juelich Histological Atlas and the JHU DTI-based WM Atlas (Eickhoff et al., 2005, 2006, 2007; Mori et al., 2005; Wakana et al., 2007; Hua et al., 2008). The 24 ROI masks were individually multiplied over both concussion and control subjects’ transformed FA, AD, and RD images. Control data ROIs were individually tested for normality using the Shapiro-Wilk statistic (*p* < 0.01) to confirm the validity of the *Z*-scoring analysis (Shapiro and Wilk, 1965).

### Statistical Analysis

For the subject-specific *Z*-score analysis each subject had 72 *Z*-scores performed (i.e., 24 brain ROIs each having measures of FA, AD, and RD):

$$Z_{ROI}=\frac{\chi_{ROI}-\mu_{C}}{\sigma_{C}}$$

where, for a particular ROI in the subject’s brain *x*_ROI_ represents the DTI metric value, μ_C_ represents the mean of the control group, and σ_C_ is the standard deviation of the control group. Typically, when conducting multiple analyses on the same dependent variable, one needs to perform a Bonferroni correction to minimize Type I errors. However, we were not performing repeated testing on the same dependent variable since each brain ROI in each subject and each DTI parameter were considered independent.

To determine whether DTI correlated with the clinically assessed injury a multiple linear regression was performed using R (v.3.6.1) and RStudio (v.1.2.1335). The number of adversely affected brain areas, as determined using DTI *Z*-scoring, was compared to total PCSS score, age at the time of injury, and time from injury to MRI assessment. This was performed as a two-tailed test with a significance threshold set at *p* < 0.05.

Even though concussions should be analyzed on a per-person basis, group analysis was done to assess whether there were brain regions where the injury occurred more commonly in the entire sample relative to healthy controls. A two-tailed Welch’s *t*-test, here with Bonferroni correction, was used to test the hypothesis that both datasets come from the same population. This statistic was chosen because it assumes both samples have normal distributions that are independent of each other, and it does not assume the variances of each sample to be equivalent (Montgomery and Runger, 2010). A significant threshold of *p* < 0.05 was used.

## Results

Based on the Shapiro-Wilk test on healthy control brains, all ROIs passed the test for normality for FA. However, two ROIs (right acoustic radiation and corpus callosum) did not meet assumptions for normality for RD and three ROIs (cingulum, corpus callosum, and uncinate fasciculus) did not meet assumptions for normality for AD (Table 2). The metrics for these specific regions were not used in the subsequent *Z*-scoring analysis.

**Table 2:** Shapiro-Wilk normality test for control brains (*n* = 49) showing the calculated W-Statistic values.

Region of Interest (ROI)	W-Statistic		
	FA	AD	RD
Acoustic Radiation Left	0.9675	0.9439	0.9634
Acoustic Radiation Right	0.9308^†^	0.9655	0.8755^¤^
Cingulate Gyrus Left	0.9684	0.9732	0.9293^†^
Cingulate Gyrus Right	0.9312^†^	0.9295^†^	0.9676
Cingulum Left	0.9545	0.8603^¤^	0.9576
Cingulum Right	0.9445	0.8391^¤^	0.9544
Corpus Callosum	0.9545	0.7990^¤^	0.7244^¤^
Corticospinal Tract Left	0.9653	0.9687	0.9295^†^
Corticospinal Tract Right	0.9373	0.9437	0.9411
Forceps Major	0.9551	0.9787	0.9301^†^
Forceps Minor	0.9680	0.9400	0.9719
Fornix	0.9291^†^	0.9327	0.9303^†^
Hippocampus Left	0.9300^†^	0.9538	0.9746
Hippocampus Right	0.9529	0.9672	0.9555
Inferior Fronto-occipital Fasciculus Left	0.9846	0.9299^†^	0.9741
Inferior Fronto-occipital Fasciculus Right	0.9679	0.9500	0.9291^†^
Inferior Longitudinal Fasciculus Left	0.9291	0.9345	0.9293
Inferior Longitudinal Fasciculus Right	0.9788	0.9615	0.9311^†^
Optic Radiation Left	0.9451	0.9635	0.9593
Optic Radiation Right	0.9385	0.9506	0.9397
Superior Longitudinal Fasciculus Left	0.9662	0.9348	0.9634
Superior Longitudinal Fasciculus Right	0.9758	0.9564	0.9756
Uncinate Fasciculus Left	0.9462	0.8777^¤^	0.9732
Uncinate Fasciculus Right	0.9589	0.8865^¤^	0.9326^†^

*Note. ^†^Denotes α > 0.01 after 2 or fewer outliers removed from data set, thus the sample was considered normally distributed. ^¤^Denotes α < 0.01 therefore reject H_0_, indicating the sample was not normally distributed*.

### Subject-Specific *Z*-Score Analysis

Subject-specific outlier detection using voxel-wise *Z*-scores from 24 brain regions in each of the concussion patients (*n* = 26) were calculated for FA, AD, and RD, where outliers identified regions of abnormality. The outlier *Z*-score distributions between left and right hemispheres were fairly even for FA (58 and 45, respectively), and RD (28 and 21, respectively). However, AD found far fewer abnormalities in the left compared to the right hemisphere (5 and 21, respectively).

Outliers in FA *Z*-score values were most frequently observed in the left inferior fronto-occipital fasciculus (12 patients), left inferior longitudinal fasciculus (12 patients), and in both the left and right superior longitudinal fasciculus (11 and 17 patients, respectively; Table 3). Fewer outliers were identified in the cingulum, cingulate gyrus, corpus callosum, fornix, and hippocampus, all having three or fewer subjects exhibiting anomalies.

**Table 3:** *Z*-score outliers for the DTI metrics of fractional anisotropy (FA), axial diffusivity (AD) and radial diffusivity (RD).

Region of Interest	Fractional anisotropy (FA)				Axial diffusivity (AD)				Radial diffusivity (RD)			
	Control Mean	Control SD	No. Outliers (± 2σ)	No. Outliers (± 3σ)	Control Mean	Control SD	No. Outliers (± 2σ)	No. Outliers (± 3σ)	Control Mean	Control SD	No. Outliers (± 2σ)	No. Outliers (± 3σ)
Acoustic Radiation Left	0.2844	0.0257	5	0	1.16 × 10^−3^	4.15 × 10^−5^	1	0	7.68 × 10^−4^	4.98 × 10^−5^	4	0
Acoustic Radiation Right	0.2724	0.0257	4	0	1.17 × 10^−3^	3.60 × 10^−5^	6	1	—	—	—	—
Cingulate Gyrus Left	0.3609	0.0379	2	0	1.12 × 10^−3^	6.49 × 10^−5^	0	0	6.47 × 10^−4^	3.71 × 10^−5^	5	0
Cingulate Gyrus Right	0.3095	0.0485	1	0	1.07 × 10^−3^	6.25 × 10^−5^	0	0	6.76 × 10^−4^	4.08 × 10^−5^	5	0
Cingulum Left	0.3455	0.0407	3	0	—	—	—	—	6.74 × 10^−4^	4.31 × 10^−5^	0	0
Cingulum Right	0.358	0.0513	0	0	—	—	—	—	6.59 × 10^−4^	4.59 × 10^−5^	0	0
Corpus Callosum	0.4076	0.0319	0	0	—	—	—	—	—	—	—	—
Corticospinal Tract Left	0.4713	0.0229	3	2	1.32 × 10^−3^	6.14 × 10^−5^	3	0	6.52 × 10^−4^	6.32 × 10^−5^	6	0
Corticospinal Tract Right	0.4652	0.0219	5	1	1.32 × 10^−3^	6.11 × 10^−5^	10	1	6.52 × 10^−4^	5.40 × 10^−5^	10	2
Forceps Major	0.3871	0.0512	0	0	1.34 × 10^−3^	9.49 × 10^−5^	3	0	7.29 × 10^−4^	7.91 × 10^−5^	1	0
Forceps Minor	0.377	0.0265	3	1	1.22 × 10^−3^	4.73 × 10^−5^	1	0	6.90 × 10^−4^	3.60 × 10^−5^	4	1
Fornix	0.2999	0.0415	2	0	1.68 × 10^−3^	1.66 × 10^−4^	2	0	1.09 × 10^−3^	1.85 × 10^−4^	1	0
Hippocampus Left	0.2652	0.0405	2	0	1.12 × 10^−3^	5.31 × 10^−5^	0	0	7.60 × 10^−4^	6.24 × 10^−5^	0	0
Hippocampus Right	0.2764	0.0399	1	0	1.13 × 10^−3^	4.88 × 10^−5^	4	0	7.60 × 10^−4^	6.22 × 10^−5^	1	0
Inferior Fronto-occipital Fasciculus Left	0.3978	0.0207	12	2	1.20 × 10^−3^	6.05 × 10^−5^	0	0	6.53 × 10^−4^	3.27 × 10^−5^	0	0
Inferior Fronto-occipital Fasciculus Right	0.3945	0.0255	5	0	1.21 × 10^−3^	5.54 × 10^−5^	0	0	6.56 × 10^−4^	4.56 × 10^−5^	0	0
Inferior Longitudinal Fasciculus Left	0.3517	0.023	12	1	1.17 × 10^−3^	6.54 × 10^−5^	0	0	6.88 × 10^−4^	3.58 × 10^−5^	0	0
Inferior Longitudinal Fasciculus Right	0.36	0.0282	6	1	1.15 × 10^−3^	5.79 × 10^−5^	0	0	6.72 × 10^−4^	4.26 × 10^−5^	0	0
Optic Radiation Left	0.2975	0.0157	1	0	1.15 × 10^−3^	4.88 × 10^−5^	1	0	7.28 × 10^−4^	3.29 × 10^−5^	2	0
Optic Radiation Right	0.3092	0.0192	2	0	1.14 × 10^−3^	4.27 × 10^−5^	1	0	7.09 × 10^−4^	3.34 × 10^−5^	0	0
Superior Longitudinal Fasciculus Left	0.3337	0.0178	11	4	1.10 × 10^−3^	4.41 × 10^−5^	0	0	6.78 × 10^−4^	2.85 × 10^−5^	1	0
Superior Longitudinal Fasciculus Right	0.3482	0.0185	17	8	1.10 × 10^−3^	4.12 × 10^−5^	0	0	6.62 × 10^−4^	3.12 × 10^−5^	1	0
Uncinate Fasciculus Left	0.3786	0.0322	7	1	—	—	—	—	6.78 × 10^−4^	3.83 × 10^−5^	10	3
Uncinate Fasciculus Right	0.3534	0.0401	4	0	—	—	—	—	7.15 × 10^−4^	5.55 × 10^−5^	4	0

*An outlier from the *Z*-distribution implies that area of the brain to be injured. Note: ROIs that failed the normality test and therefore, could not be used for analysis are signified by “—“*.

For RD outliers, the right corticospinal tract and left uncinate fasciculus were both frequently classified as outliers (10 of 26 patients; Table 3). For both, every outlier had *Z* > 2. In the corticospinal tract *Z* > 0 for 22 of the 26 subjects and in the uncinate fasciculus *Z* > 0 for 25 of the 26 subjects indicated that RD increased in these regions for most concussion subjects relative to healthy controls.

Outliers in AD identified the least number of concussion subjects brain differences compared to controls (Table 3). The right corticospinal tract was the only region that had a significant number of abnormalities (10 of 26 patients). All 10 patients had *Z* > 2, and 25 of the 26 subjects had *Z* > 0. Of note, nine of the 19 normally distributed regions did not produce any outliers.

**Figure 1:**
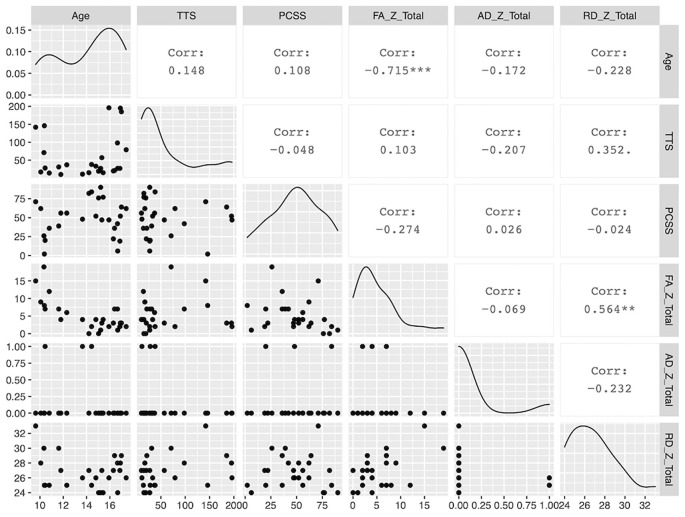
A paired matrix plot indicating the distribution of data for each metric and the correlation between metrics.

**Figure 2:**
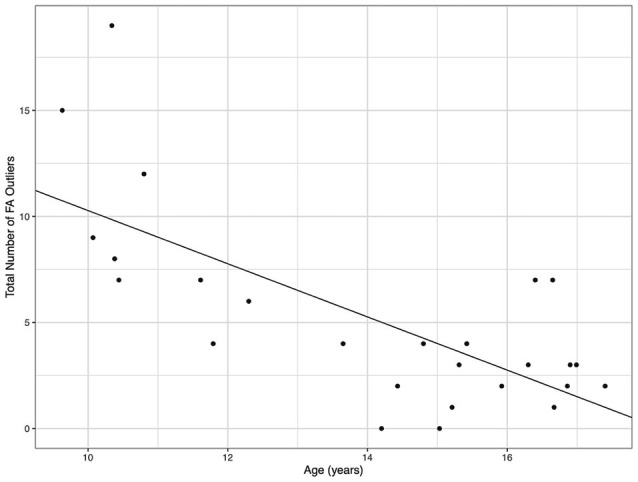
The total number of fractional anisotropy (FA) outliers against the age for each concussion subject. A greater number of FA outliers were found in younger subjects.

**Figure 3:**
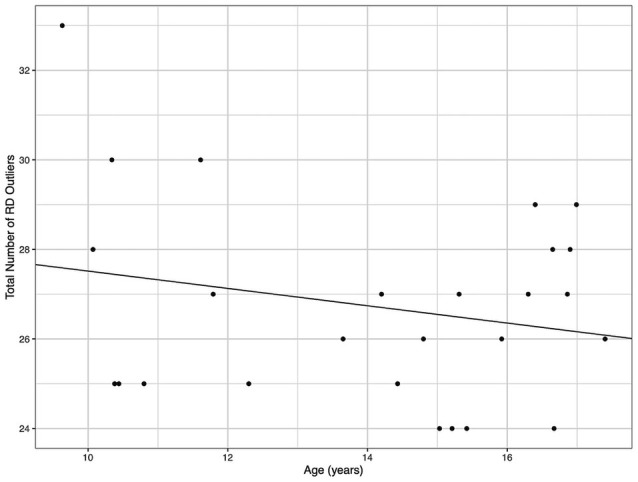
The total number of radial diffusivity (RD) outliers against the age for each concussion subject. A greater number of RD outliers were found in younger subjects.

Demographic parameters were significantly correlated with DTI metrics (Figure 1). The strongest and most significant correlations were with FA (Adjusted R-squared: 0.5725, *p* < 0.001) and RD (Adjusted R-squared: 0.3295, *p* = 0.013; Table 4). Specifically, total FA injury burden was significantly and negatively associated with age at injury (*p* < 0.001), which indicated that younger subjects experienced greater WM damage (Figure 2). There was a very weak negative correlation between FA-based disease burden and PCSS (correlation coefficient = −0.274, *P* < 0.038). RD injury burden was also significantly correlated with age at injury (*p* = 0.029; Figure 3) and the time to scan (TTS) × PCSS interaction (*p* = 0.0078).

**Table 4:** Results of the multiple linear regression for the three DTI metrics against the patient demographic information of patient age, the time from their concussion to the MRI scan (TTS), Post-Concussion Symptom Scale (PCSS) score and the interaction between TTS and PCSS score.

DTI Metric	Coefficient	Estimate	Std. Error	*t*-value	Pr (>|t|)
FA	Intercept	27.474	3.985	6.894	<0.001***
	Age	−1.362	0.239	−5.691	<0.001***
	TTS	−0.0281	0.0271	−1.038	0.311
	PCSS	−0.0809	0.0365	−2.214	0.038*
	TTS:PCSS	8.76 × 10^−4^	5.1 × 10^−4^	1.718	0.101
AD	Intercept	0.515	0.456	1.130	0.271
	Age	−0.0211	0.0274	−0.771	0.449
	TTS	−0.00223	0.00310	−0.720	0.480
	PCSS	0.00418	−0.205	0.839
	TTS:PCSS	2.53 × 10^−5^	5.83 × 10^−5^	0.433	0.669
RD	Intercept	32.923	2.419	13.609	<0.001***
	Age	−0.341	0.145	−2.344	0.0290*
	TTS	−0.0306	0.0164	−1.859	0.0771.
	PCSS	−0.0446	0.0222	−2.011	0.0573.
	TTS:PCSS	9.11 × 10^−4^	3.1 × 10^−4^	2.941	0.0078**

Note: Significance codes: 0 ‘***’; 0.001 ‘**’; 0.01 ‘*’; 0.05 ‘.’; 0.1 ‘ ’ 1. For FA: Residual standard error: 2.994 on 21 degrees of freedom. Multiple R-squared: 0.6409, Adjusted R-squared: 0.5725. F-statistic: 9.368 on 4 and 21 DF, p-value: 0.0001653. For AD: Residual standard error: 0.3423 on 21 degrees of freedom. Multiple R-squared: 0.07259, Adjusted R-squared: −0.1041. F-statistic: 0.4109 on 4 and 21 DF, p-value: 0.7988. For RD: Residual standard error: 1.817 on 21 degrees of freedom. Multiple R-squared: 0.4367, Adjusted R-squared: 0.3295. F-statistic: 4.071 on 4 and 21 DF, *p*-value: 0.013473.

### Group Z-Score Analysis

Even though concussions are best assessed individually, group analysis was carried out to identify whether any particular brain regions more frequently appeared as abnormal post-concussion. As with *Z*-scoring, *t*-tests also require the data to follow a Gaussian distribution. All FA comparisons met the assumptions for normality. The concussion group was significantly different in FA from controls in 19 of the 24 ROIs analyzed. The remaining five regions that were not significantly different from controls (*p* > 0.05) were the left and right cingulum, corpus callosum, forceps major, and fornix. Additionally, in every ROI, excluding the forceps major, the mean FA for the concussion group was less than for the control group.

For RD, the concussion group was significantly different from the control group in 14 of the 22 normally distributed, testable ROIs. The corticospinal tract and uncinate fasciculus regions had the greatest probability of being from two different populations with *p*-values < 0.001. The mean RD of the concussion group was greater than controls in 15 of the 22 regions tested. Similarly, RD was greater for the concussion group in 10 of the 14 significantly different regions. The regions whose AD and RD values were not normally distributed (five regions for AD, and two for RD) were not analyzed.

For AD, significant differences between the concussion and control groups were found in 9 of the 19 normally distributed, testable ROIs. Of those that passed normality testing, the following appeared significantly different unilaterally: the acoustic radiation, hippocampus, inferior fronto-occipital fasciculus, and inferior longitudinal fasciculus. However, both left and right corticospinal tracts and left and right optic radiation regions showed the highest degree of significant difference between concussion and control data with all of their *p*-values <0.001. In 11 of the 19 regions, the concussion group had a higher mean AD than the control group. A similar pattern was observed among the nine significantly different ROIs with greater mean AD values found in five of those regions.

## Discussion

The important finding from this work was that a concussion could be assessed in an individual paediatric subject using *Z*-scored DTI metrics. The three primary metrics (FA, AD, and RD) may not uniformly show brain ROI abnormalities, but together can provide an understanding of the global structural abnormalities unique to a subject following a concussion. Furthermore, although the assumption of normality must be confirmed, comparing individuals with a concussion to healthy controls is a viable technique since the large normative healthy dataset is often normally distributed.

A number of previous studies have reported lower FA values and higher RD and AD measurements when analyzing concussion relative to age-matched control groups using group-based statistics (Inglese et al., 2005; Cubon et al., 2011). This study aimed to build upon past research by investigating the efficacy of a novel approach to improve individualized concussion identification. It was hypothesized that FA would be decreased in the concussion group relative to healthy controls, and within our concussion subjects, 19 of the 24 ROIs analyzed showed lower values in at least one individual (Table 3). The regions not showing significantly lower *Z*-scores were the deep brain regions (left and right cingulum, corpus callosum, forceps major, and fornix). Many have suggested, albeit based on group analysis, that the corpus callosum would be a significant focus for damage in mTBI due to stresses and shearing forces (Kinnunen et al., 2011). Although corpus callosum FA was reduced for concussion subjects in this study, it was not significantly different from control values. It was also hypothesized that concussion would result in increased RD and AD. But these measures were more variable than that seen with FA, showing seven ROIs that were significantly elevated for both metrics; the corticospinal tract, forceps minor, right hippocampus, left inferior longitudinal fasciculus, and optic radiation. The left and right corticospinal tracts saw the most dramatic differences between the two groups, with approximately 11% greater RD and 6% greater AD. The group analysis had variable efficacy in identifying abnormalities in a single mTBI patient, relative to controls, in terms of FA, RD, and AD measurements. In only some instances did all three metrics detect abnormalities in the same brain region in the same patient. Table 5 provides a rank-order of these seven ROIs based on each region’s average *p*-value calculated for FA, AD, and RD (where *p*avg = [*p*_FA_+*p*_AD_+*p*_RD_]/3). This data suggests that the corticospinal tract was the most effective region to differentiate between the mTBI and healthy control groups.

**Table 5:** Relative difference between the mTBI and control groups for AD and RD, and the average *p*-value across all DTI metrics for only the ROIs that satisfied *p* < 0.05 for both AD and RD metrics.

	% Difference (mTBI vs. Control)		Average *P*-value (FA + AD + RD)/3
Region of Interest (ROI)	AD	RD	
Corticospinal Tract Left	5.5%	11.3%	1.53 × 10^−7^
Corticospinal Tract Right	6.6%	11.2%	5.52 × 10^−6^
Forceps Minor	2.6%	5.5%	9.35 × 10^−5^
Hippocampus Right	2.4%	3.9%	9.19 × 10^−4^
Inferior Longitudinal Fasciculus Left	−3.1%	−2.0%	3.75 × 10^−3^
Optic Radiation Left	−4.5%	−4.5%	7.55 × 10^−3^
Optic Radiation Right	−3.8%	−3.1%	1.43 × 10^−2^

One key factor that is often overlooked in MRI studies of concussion is its correlation with clinical metrics. The PCSS score is the most frequently used clinical tool. As it is not possible to correlate any one PCSS symptom with one brain ROI, overall lesion burden (i.e., number of abnormal ROIs per metric per subject) was used to compare against PCSS, age at injury, and time to scan (TTS). It was found that younger age was a strong predictor (correlation coefficient = −0.715, *P* < 0.001) of more MRI indicated damage. This suggests that age plays a role in brain injuries and must be considered during the diagnosis as older children had fewer outlier regions. As paediatric brains are still developing the influence of a concussion may be more severe than that experienced by an adult brain (Zuckerman et al., 2012). Although PCSS was significantly correlated with FA injury burden, it was calculated to have a very weak, negative correlation. The PCSS remains a useful diagnostic tool to assess concussion recovery, however its accuracy and subjectivity when used for youth may explain these results. Further research with a larger sample size is required to draw final conclusions on the link between MRI and PCSS compatibility.

The FA measurements identified significant abnormalities in the inferior and superior longitudinal fasciculus and the inferior fronto-occipital fasciculus. These are longer WM tracts and are possibly more vulnerable to rotational and shearing forces. A number of imaging studies have also identified microstructural damage in the superior longitudinal fasciculus post-mTBI (Kraus et al., 2007; Morey et al., 2013; O’Phelan et al., 2018). Involved in auditory, visual, and memory integration, impairment within these regions may decrease the ability to comprehend and remember new information, both written and spoken, which is common with concussions. The uncinate fasciculus was found abnormal for a number of subjects, based on RD, and the corticospinal tract was greatly impacted in terms of both RD and AD. The uncinate fasciculus’ primary function is memory integration with functional importance to the integration of social and emotional behavior (Von der Heide et al., 2013). The corticospinal tract processes motor control by conducting brain impulses to the spinal cord. Damage to the uncinate fasciculus and corticospinal tracts would include frequently reported post-concussion symptoms such as auditory, memory, and motor impairments. Previous work supports our findings where only the longitudinal fasciculus and corticospinal tracts had axonal damage in concussion (Wakana et al., 2007; Cubon et al., 2011), while 13 brain regions saw reductions in FA for moderate to severe TBI. Few ROIs, including the corpus callosum, were not significantly different from control data regardless of the metric. A past study from Morey and colleagues showed correlation with concussion severity and WM integrity in the corpus callosum and other deeper brain structures (Mori et al., 2005; Eickhoff et al., 2007; Morey et al., 2013). Although these findings are in disagreement with this study, possibly because they performed group rather than individual analysis, it should be noted that FA was the only testable metric in this study for the corpus callosum because corpus callosum RD and AD data failed the normality test and therefore no further analysis could be performed (Table 2).

Overall, it was confirmed that the suggested loss of WM integrity, measured through changes in DTI metrics, could be quantified on a personalized basis. Using large normative data, we were able to identify significant regional brain abnormalities on an individual basis. Because the three DTI metrics (FA, RD, and AD) did not always corroborate, more advanced imaging approaches such as high angular resolution diffusion imaging (HARDI) and Diffusion Kurtosis Imaging (DKI) with even larger normative datasets are unquestionably necessary for future research. The more advanced DWI techniques of HARDI and DKI, and their subsequent analyses such as neurite orientation dispersion and density imaging (NODDI) or constrained spherical deconvolution (CSD), can provide more accurate estimations of voxel properties because they utilize more diffusion directions and higher b-values than DTI (Danielli et al., 2020). Thus, making HARDI and DKI more accurate than DTI as they are able to measure multiple fiber directions, orientations, densities, and dispersions within voxels because they are not limited to Gaussian distributions (Danielli et al., 2020). As seen in Table 3 of our study, a large number of FA outliers were calculated in several brain regions with no outliers calculated for AD or RD (i.e., the inferior fronto-occipital fasciculus left (FA = 12, AD = 0, RD = 0), inferior longitudinal fasciculus left (FA = 12, AD = 0, RD = 0), superior longitudinal fasciculus left (FA = 11, AD = 0, RD = 1) and superior longitudinal fasciculus right (FA = 17, AD = 0, RD = 1). Although FA appeared to be the most sensitive DTI metric there was still value in exploring the diffusion metrics of AD and RD as outliers were detected in other ROIs, and RD showed significant correlations with demographic factors. The discrepancy between metrics may also have been the result of DTI limitations where HARDI or DKI acquisitions might have detected outliers due to their more accurate estimates. Nonetheless, consideration of diffusion metrics beyond FA can provide insightful information and should be implemented more often in research. In conclusion, this study demonstrated that DTI can sensitively detect microstructural changes caused by a concussion and has the potential to be an effective tool to accurately diagnosis concussions on a person-by-person basis.

## Data Availability Statement

The raw data supporting the conclusions of this article will be made available by the authors, upon request.

## Ethics Statement

The studies involving human participants were reviewed and approved by Hamilton Integrated Research Ethics Board (HiREB). Written informed consent to participate in this study was provided by the participants’ legal guardian/next of kin.

## Author Contributions

Experimental design and planning: MN, CD, JC, GH, and NB. MRI scanning: DS, RH, and MN. Writing of manuscript: DS, ED, and RH. Statistics: DS and ED. Patient recruitment: CD and RH. Original concept of work: MN and CD. Manuscript revisions and edits: ED, DS, RH, MN, CD, and JC. Acquisition of funding: MN, CD, JC, GH, and NB. All authors contributed to the article and approved the submitted version.

## Conflict of Interest

MN is the Co-Founder and CEO of TBIfinder, a data analytics company. No technology or funding from TBIfinder was contributed to this submitted study. The remaining authors declare that the research was conducted in the absence of any commercial or financial relationships that could be construed as a potential conflict of interest.
